# Ubiquitous miR159 repression of *MYB33*/*65* in Arabidopsis rosettes is robust and is not perturbed by a wide range of stresses

**DOI:** 10.1186/s12870-016-0867-4

**Published:** 2016-08-19

**Authors:** Yanjiao Li, Maria Alonso-Peral, Gigi Wong, Ming-Bo Wang, Anthony A Millar

**Affiliations:** 1 Plant Science Division, Research School of Biology, Australian National University, Canberra, 2601 ACT Australia; 2 CSIRO, Agriculture, Canberra, 2601 ACT Australia

**Keywords:** miR159, *GAMYB-like*, Arabidopsis, Stress, Viral silencing suppressors

## Abstract

**Background:**

The microR159 (miR159) – *GAMYB* pathway is conserved in higher plants, where *GAMYB*, expression promotes programmed cell death in seeds (aleurone) and anthers (tapetum). In cereals, restriction of *GAMYB* expression to seeds and anthers is mainly achieved transcriptionally, whereas in Arabidopsis this is achieved post-transcriptionally, as miR159 silences *GAMYB* (*MYB33* and *MYB65*) in vegetative tissues, but not in seeds and anthers. However, we cannot rule out a role for miR159-*MYB33*/*65* pathway in Arabidopsis vegetative tissues; a loss-of-function *mir159* Arabidopsis mutant displays strong pleiotropic defects and numerous reports have documented changes in miR159 abundance during stress and hormone treatments. Hence, we have investigated the functional role of this pathway in vegetative tissues.

**Results:**

It was found that the miR159-*MYB33*/*65* pathway was ubiquitously present throughout rosette development. However, miR159 appears to continuously repress *MYB33*/*MYB65* expression to levels that have no major impact on rosette development. Inducible inhibition of miR159 resulted in *MYB33*/*65* de-repression and associated phenotypic defects, indicating that a potential role in vegetative development is only possible through *MYB33* and *MYB65* if miR159 levels decrease. However, miR159 silencing of *MYB33*/*65* appeared extremely robust; no tested abiotic stress resulted in strong miR159 repression. Consistent with this, the stress responses of an Arabidopsis mutant lacking the miR159-*MYB33*/*65* pathway were indistinguishable from wild-type. Moreover, expression of viral silencing suppressors, either via transgenesis or viral infection, was unable to prevent miR159 repression of *MYB33*/*65*, highlighting the robustness of miR159-mediated silencing.

**Conclusions:**

Despite being ubiquitously present, molecular, genetic and physiological analysis failed to find a major functional role for the miR159-*MYB33*/*65* pathway in Arabidopsis rosette development or stress response. Although it is likely that this pathway is important for a stress not tested here or in different plant species, our findings argue against the miR159-*MYB33*/*65* pathway playing a major conserved role in general stress response. Finally, in light of the robustness of miR159-mediated repression of *MYB33*/*65*, it appears unlikely that low fold-level changes of miR159 abundance in response to stress would have any major physiological impact in Arabidopsis.

**Electronic supplementary material:**

The online version of this article (doi:10.1186/s12870-016-0867-4) contains supplementary material, which is available to authorized users.

## Background

The miR159 family represents one of the most ancient miRNA families being present in most land plants (>400 million years) [1]. Via bioinformatic prediction and experimental validation, miR159 has been found to regulate the expression of a family of *GAMYB* or *GAMYB*-like genes in a diverse range of plant species, including monocots such as barley and rice [2], dicots such as *Arabidopsis* [3], potato [4] and strawberry [5], and gymnosperms such as Larix [6]. Despite the considerable evolutionary distance that separates these species, the miR159 binding site in these *MYB* genes is conserved in both position and sequence, inferring this miR159-*MYB* relationship has a long co-evolutionary history. This strong conservation indicates this miR159-*MYB* relationship has been under strong selective pressure, presumably performing a critical function.

These *GAMYB* genes encode R2R3 MYB domain transcription factors that have been implicated in gibberellin (GA) signal transduction. Their role has been best characterised in anthers (tapetum) and seeds (aleurone), where a major role is to promote programmed cell death (PCD). In rice, a loss-of-function *gamyb* mutant is male sterile as the tapetum fails to undergo PCD and degenerate [7, 8]. Likewise in *Arabidopsis*, mutation of *MYB33* and *MYB65*, the two major target genes of miR159, results in male sterility due to a tapetum that fails to degenerate [9, 10]. Supporting these observations is the overexpression of miR159 in both cereals and Arabidopsis which results in male sterility [2, 11–13], implying this GAMYB anther function has been strongly conserved. In the seed, GAMYB expression in barley or *Arabidopsis* promotes aleurone vacuolation, also a GA-mediated PCD process [14, 15]. Therefore, it appears that in seeds where miR159 activity is weak [2, 16], these *GAMYB-like* genes are expressed, promoting a conserved PCD function.

By contrast, the functional role of the miR159-*MYB* pathway in vegetative tissues is not as clear. A role for miR159 in development has been suggested from genetic analysis in Arabidopsis. Previously, loss-of-function mutations have been isolated for all three Arabidopsis miR159 family members, miR159a-c. None of the three single mutants displayed a phenotype, but a *mir159ab* double mutant displayed pleiotropic developmental defects, that included stunted growth and rounded, upwardly curled leaves [10, 17]. This was consistent with deep sequencing; miR159a and miR159b were found to be overwhelmingly the major isoforms, composing approximately 90 % and 10 % of the miR159 reads respectively in Arabidopsis [18]. By contrast, miR159c is very lowly expressed, and there are multiple lines of evidence indicating this miRNA is likely non-functional in Arabidopsis [17]. Although miR159 is predicted to regulate approximately 20 different target genes in Arabidopsis, all the *mir159ab* pleiotropic vegetative defects are suppressed in a *mir159ab.myb33.myb65* quadruple mutant, genetically demonstrating that miR159 is functionally specific for *MYB33* and *MYB65* in vegetative tissues, although this does not dismiss the possibility of miR159-mediated regulation of other targets that do not result in obvious developmental defects. Nevertheless, partly explaining this specificity is that many of the other potential miR159 targets are not transcribed in vegetative tissues, but rather their transcription is restricted to anthers [17]. Together, these experiments have defined a highly active miR159-*MYB33*/*MYB65* pathway present in Arabidopsis vegetative tissues.

Curiously, rosettes of a *myb33.myb65* double mutant have no major phenotypic defects, where multiple lines of evidence suggest that miR159-mediated silencing represses the expression of these genes to low levels [15], raising the question of what functional role this pathway plays in rosettes. Although a role for *MYB33* in promoting flowering has been proposed, as miR159 overexpression in the Landsberg *erecta* ecotype reduced *MYB33* transcript levels and delayed flowering [11], no such impact was seen in the Columbia ecotype [12]. Furthermore, flowering was neither delayed in *myb33.myb65* nor promoted in *mir159ab* indicating *MYB33/MYB65* are not major players in determining flowering-time in Columbia [15]. Therefore, no clear rationale exists for this miR159-*MYB33*/*MYB65* pathway in rosette/vegetative tissues, at least under standard growing conditions.

Interestingly, similar to many other highly conserved miRNAs, numerous studies that have quantified changes to miRNA levels have implicated miR159 in playing a response to a variety of stresses in a number of different species. This includes drought [4, 19], salinity [20], cold [21], heat [13], UV-light [22], waterlogging [23] or response to biotic stresses such as viruses [24, 25] or bacterial lipopolysaccharides [26]. Given MYB activity can impact vegetative growth its expression may adjust growth during stress [20]. However, no clear trend in miR159 abundance emerges from these stress treatments, where in some instance miR159 abundance increases with stress treatment [19, 22, 23, 26], and in others, miR159 abundance decreases [4, 13]. Whether these changes result in significant physiological responses to these stresses and whether any potential role is widely conserved is unclear. Therefore, despite the miR159-*MYB* being strongly conserved across many species, what functional role this pathway plays in vegetative tissues remains unknown. To address this question, we have attempted to determine in what developmental stages of the rosette development are miR159 and *MYB33*/*65* expressed/transcribed. We then investigate under what conditions miR159 is sufficiently suppressed enabling de-repression of *MYB33/65* expression, and whether this alteration in miR159 contributes to a physiological response to the stress. Such experiments will help determine what functional role the miR159-*MYB* pathway performs in vegetative tissues of *Arabidopsis*.

## Results

### The miR159-*MYB33*/*65* module is ubiquitously present in *Arabidopsis* rosettes

**Fig. 1:**
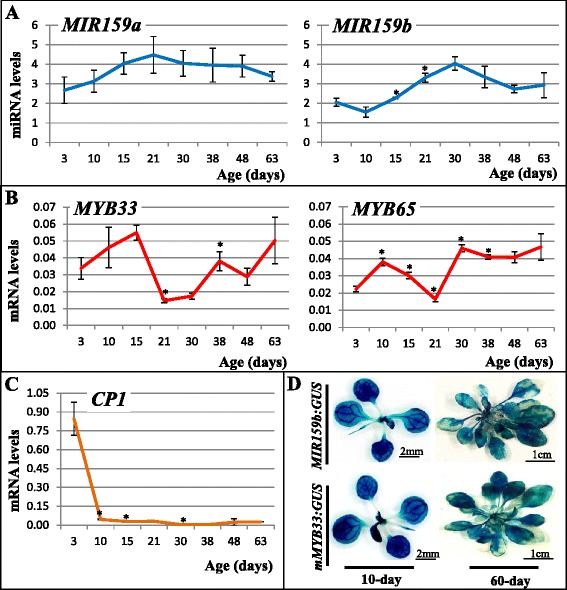
MiR159 constitutively silences *MYB33/65* throughout rosette development. **a**–**c** Time-course transcript profiling of the miR159-*MYB* pathway in rosettes. The relative miRNA and mRNA levels were measured in rosettes approximately every 10 days throughout its development. The miR159 levels were normalized to *sno101*, the mRNA levels were normalized to *CYCLOPHILIN*. Values are the mean of three technical replicates with error bars representing the Standard Deviation (SD). Significant differences in values from the previous measurement are indicated with an _*_, as determined by the Students *T*-test. **d** Time-course GUS-staining assay for rosettes of *MIR159b:GUS* and *mMYB33:GUS* transgenic lines. Staining was carried out on ten individual rosettes per time point, at ten-day interval during plant growth; only the first and last staining results are shown

To begin the characterisation of miR159-*MYB* pathway in *Arabidopsis* rosettes, two time-course experiments were performed to determine in what developmental stages and rosette tissues miR159 and *MYB33/65* are expressed. Firstly, a qRT-PCR based transcript profiling was performed on a time-course of *Arabidopsis* rosettes grown over 60 days to determine the abundance of mature miR159a/miR159b and the mRNA levels of *MYB33/MYB65* (Fig. 1a). However, *MYB33*/*65* mRNA levels are not accurate indicators of their protein expression level due to the presence of a strong miR159-mediated translational repression mechanism [27]. Therefore, we investigated whether the mRNA levels of a downstream gene, *CYSTEINE PROTEINASE1* (*CP1*; At4g36880) [15] would be an accurate indicator of MYB33/65 activity. We found *CP1* mRNA levels tightly correlate with *MYB33* and *MYB65* mRNA levels in the absence of miR159 (the *mir159ab* mutant background; Additional file 1: Figure S1). Therefore, *CP1* levels are used throughout the study as an indicator of MYB33/MYB65 activity.

It was found that both miR159a and miR159b were expressed throughout rosette development. Both miRNAs had similar developmental profiles, increasing approximately two-fold during the first half of rosette development and then decreasing slightly (Fig. 1a). Although for miR159a, there were no significant differences in miRNA levels at the different time points, independent time-courses confirmed this pattern and the approximate two-fold increase in miR159a abundance (Additional file 2: Figure S2). For *MYB33* and *MYB65*, their transcript levels fluctuated throughout rosette development. However, their levels did not inversely correlate with the miR159 profile, and so these observed differences are likely to be independent of regulation by miR159 (Fig. 1b). Additionally, these wild-type *MYB33*/*MYB65* transcript levels are approximately 10-fold lower compared to levels in 35-day-old *mir159ab* rosettes (Additional file 1: Figure S1), suggesting *MYB33*/*MYB65* are being repressed throughout vegetative development. Supporting this notion, mRNA of the *CP1* marker gene remains low throughout wild-type rosette development (Fig. 1c), as *CP1* mRNA in 35-day-old *mir159ab* rosettes is at least an order of magnitude higher (Additional file 1: Figure S1). In *mir159ab* rosettes, the *CP1* mRNA abundance is similar to that of three-day-old wild-type seedlings, tissue that is known to have high MYB protein activity due to weak miR159 activity in seeds [15, 16]. Based on all the above data, *MYB33* and *MYB65* mRNA is likely being continually repressed throughout wild-type rosette development, and that the fluctuations observed in their transcript levels may not have any functional significance.

To determine in what rosette tissues *MIR159* and its target *MYB* genes are transcribed, a β-glucuronidase (GUS)-staining assay was carried out over a 60-day time course on two transgenic *Arabidopsis* lines; *MIR159b:GUS* and *mMYB33:GUS*. The *MIR159b:GUS* line was constructed by fusing the *GUS* gene downstream of the *MIR159b* promoter, to visualize the transcriptional domain of *MIR159b* [10], while the *mMYB33:GUS* line carries a miR159-resistant version of *MYB33*, which enables visualization of the *MYB33* transcriptional domain [9]. The rosettes of each line were harvested and stained every ten days. It was found that the rosettes of both lines stained at all the tested time points, from young seedling (10-day-old) to the late reproductive (60-day-old) growth phases (Fig. 1d). Moreover, the staining appeared ubiquitous throughout *MIR159b:GUS* and *mMYB33:GUS* rosettes. Patches of unstained cells in the older plants did not reflect a developmental pattern, but rather appeared to correspond to dead cells or a leaf staining penetration problem. Hence, both *MIR159b* and *MYB33* appear transcribed in all cells and at all rosette developmental stages. Together, the data suggests the strong constitutive expression of miR159 that suppresses the expression of the constitutively transcribed *MYB33* and *MYB65* genes throughout *Arabidopsis* rosette development.

### MiR159 is functionally active throughout rosette development

**Fig. 2:**
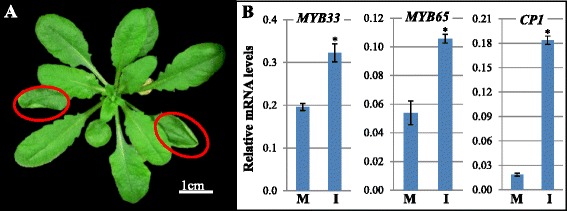
Morphological and molecular phenotypes via induced inhibition of miR159. **a** Application of 17-β-estradiol to 21-day-old *XVE-MIM159* transformants induced leaf-curling (red circled). The representative picture was taken when plants were 35-day-old short-day grown plants. **b** qRT-PCR of *MYB33*, *MYB65* and *CP1* mRNA levels in 35-day-old *XVE-MIM159* rosettes with either mock (M) or inducer (I) treatments. mRNA levels were normalized to *CYCLOPHILIN*. Values are the mean of three technical replicates with error bars representing the SD. Significant differences in values from the untreated are indicated with an _*_

Since *35S-miR159* Arabidopsis plants have no obvious vegetative defects [12] and miR159 appears to constantly silence *MYB33* and *MYB65* under normal growth conditions (Fig. 1), a major impact of the miR159-*MYB* pathway in the rosette only appears possible if miR159 levels can be decreased enabling MYB33/MYB65 expression. To test this idea, a *XVE-MIM159* transgene was transformed into *Arabidopsis* (Fig. 2a). *XVE* is a transactivator that can be induced by estrogen (e.g. 17-β-estradiol), resulting in transcriptional activation of the downstream transgene [28], while the *MIM159* gene carries a non-cleavable miR159 binding site that inhibits miR159 function [29]. Primary *XVE-MIM159* transformants were selected and grown for 21 days so that rosettes were well established, and were then treated with either 10 μM 17-β-estradiol (inducer) or dimethyl sulfoxide (DMSO; dissolving solution, negative control). After two weeks of 17-β-estradiol treatment, rosettes contained upwardly curled leaves (Fig. 2a). This occurred in the older leaves of the plant, consistent with the older leaves of *mir159ab* displaying the strongest curl. Such phenotypes were not observed in *XVE-MIM159* transformants treated with just DMSO or in any wild-type plants grown under our conditions. Additionally, *MYB33*, *MYB65* and *CP1* mRNA levels were elevated in 17-β-estradiol treated *XVE-MIM159* plants (Fig. 2b). Together, this data suggests that miR159 function is constantly active in developing rosettes and perturbation of this function results in de-repression of *MYB33/65* expression accompanied with morphological alterations to the rosette. This raises the possibility that the miR159-*MYB* module may be involved in response to environmental stress(es), where repression of miR159 by a stress may potentially activate *MYB33/65* expression, resulting in morphological alterations in response to the stress.

### The miR159-*MYB33*/*MYB65* pathway has no major impact in the response to common abiotic stresses

MiR159 function has been implicated in plant response to abiotic stresses, as miR159 levels are repressed during heat stress in wheat [13] or in response to drought stress in potato [4]. For Arabidopsis, we searched the GENEVESTIGATOR platform (https://www.genevestigator.com/gv/) and eFP Browser (http://bar.utoronto.ca/efp/cgi-bin/efpWeb.cgi) for growth conditions that may activate *CP1* transcription based on the assumption that this gene will be activated if miR159 function is compromised. However, *CP1* mRNA levels were found to remain low under all examined growth conditions and stresses (data not shown). Hence, it is unclear whether the miR159-*MYB33*/*MYB65* pathway is playing a major role in response to abiotic/biotic stresses in *Arabidopsis*.

To investigate whether miR159 is responsive to abiotic/biotic stresses in *Arabidopsis*, wild-type plants were grown under several common environmental stresses including ABA application, heat, high light intensity, drought, and cold and miR159 levels were measured. Additionally, *mir159ab.myb33.myb65* quadruple mutant plants were grown alongside to determine whether there are alterations to growth when the miR159-*MYB33*/*65* pathway is mutated. Also included in the analysis was the loss-of-function *mir159a* mutant in which miR159 abundance is reduced to approximately 10 % of wild type levels, but is morphologically indistinguishable from wild-type [10], possibly making such a genotype sensitized to subtle perturbations of miR159 function which may not manifest in wild-type plants.

**Fig. 3:**
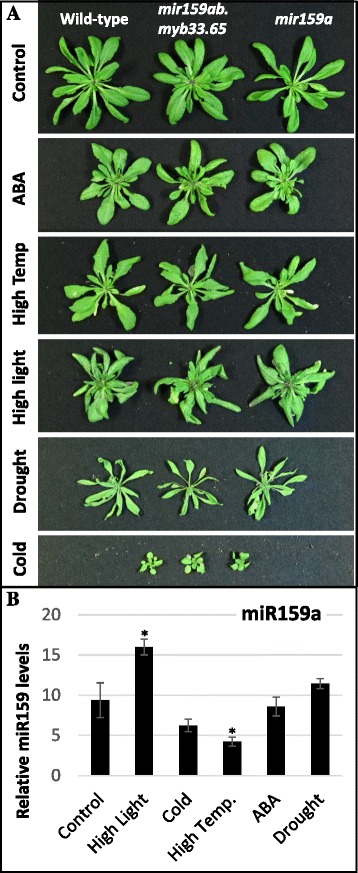
Morphological and molecular analysis of stressed *Arabidopsis* miR159-*MYB* pathway mutants. **a** Phenotypic comparison of rosettes of Col, *mir159ab.myb33.myb65* and *mir159a* plants treated with ABA, high temperature, high light, drought and cold. Plants were grown for two weeks at 21 °C and then subjected to three weeks of stress treatment (**b**) Taqman microRNA assays measuring miR159a levels in wild-type plants subjected to the above treatments. Levels were normalized to *sno101.* Values are the mean of three technical replicates with error bars representing the SD. Significant differences in values from the control are indicated with an _*_

**Fig. 4:**
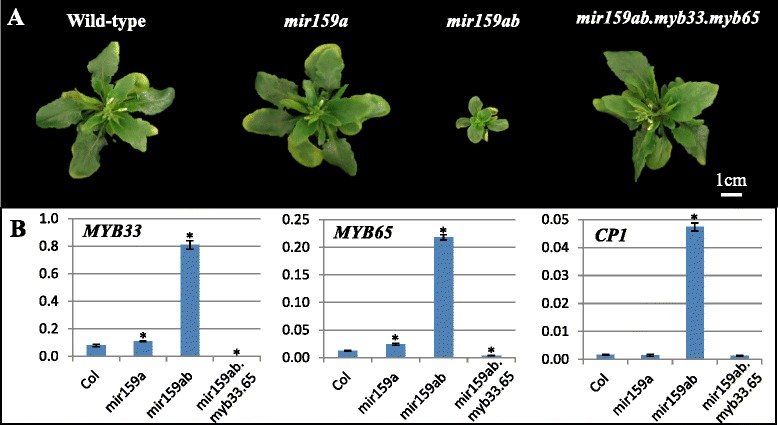
Morphological and molecular analysis of low-temperature effect on the *Arabidopsis* miR159-*MYB* pathway mutants. **a** Phenotypic comparison of rosettes of Col, *mir159a*, *mir159ab* and *mir159ab.myb33.myb65* plants stressed with low-temperature. Plants were grown for three weeks at 21 °C and then grown for eight weeks at 4 °C. **b** qRT-PCR analysis of *MYB33*, *MYB65* and *CP1* mRNA levels in the above rosettes. The mRNA levels were normalized to *CYCLOPHILIN*. Values are the mean of three technical replicates with error bars representing the SD. Significant differences in values from wild-type is indicated with an _*_

None of the tested stress conditions induced a major or obvious observable phenotypic difference between wild-type (Col), *mir159a* and *mir159ab.myb33.myb65* plants, where the two mutant genotypes appear indistinguishable from wild-type when grown under stress conditions (Fig. 3a). Consistent with this, none of the stress conditions, or the stress-related ABA hormone, resulted in suppression of miR159 to levels in which *MYB33*/*65* expression would be predicted to be de-repressed (Fig. 3b). MiR159 levels in plants subjected to high temperature had the lowest relative miR159 abundance, but this appears due to a higher level of the normalizing reference gene (*sno101*) rather than a decrease in abundance of miR159 (Additional file 3: Figure S3). Therefore, of the different conditions, low-temperature treatment appeared to result in the lowest levels of miR159, although this reduction was not statistically different from untreated (Fig. 3b). Nevertheless, we investigated miR159 response in cold stress further (Fig. 4). However, the mRNA levels of *CP1* remained unchanged between Col and the sensitized background, *mir159a* (Student’s Test: *P* > 0.05) (Fig. 4b), indicating that miR159-mediated silencing of *MYB33*/*65* had not been strongly perturbed. This supports the observation that the Col, *mir159a* and *mir159ab.myb33.myb65* plants displayed no strong morphological differences under low temperature stress (Fig. 4a). From these experiments, it appears that the miR159-*MYB33*/*65* pathway plays no major role resulting in an obvious phenotypic impact in response to these common abiotic stress conditions.

### Expression of viral silencing suppressors failed to strongly inhibit miR159

**Fig. 5:**
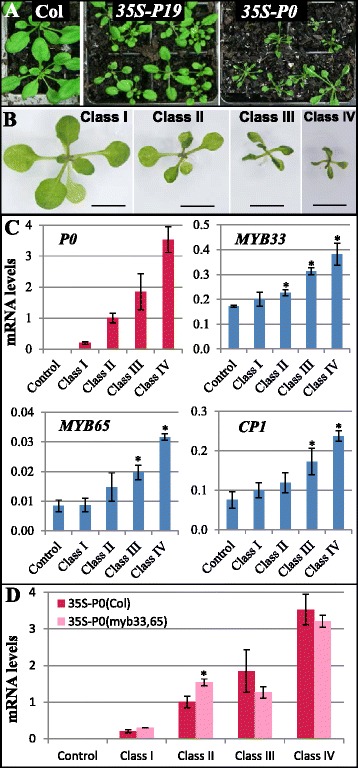
Constitutive expression of VSSs does not strongly perturb the miR159 silencing of *MYB33*/*65*. **a** Different phenotypes developed in 28-day-old *35S*-*P19* and *35S*-*P0* primary transformants with wild-type (Col) grown alongside as a control. **b** The representative classification of developmental defects among *35S*-*P0* primary transformants. Class I: wild-type-looking; Class II: mild reduction in rosette size and partially curled leaves; Class III: all leaves curled group; Class IV: severely stunted and all leaves curled. **c** qRT-PCR analysis of relative mRNA levels in the different classes. Significant difference in values from the control is indicated with an _*_. **d** Comparison of *P0* mRNA levels between *35S*-*P0*(Col) and *35S*-*P0*(*myb33.myb65*) with the same classified phenotypes. The RNA samples were extracted from 26-day-old plants. Col and *myb33.myb65* were used as controls. *P0* mRNA levels were normalized to *UBIQUITIN* (At4g05320), while *MYB33*/*65* and *CP1* were normalized to that of *CYCLOPHILIN*. Values are the mean of three technical replicates with error bars representing the SD. Significant differences between 35S-P0(Col) and 35S-P0(myb33,65) values are indicated with an _*_

One likely biotic factor that could inhibit miR159 is the expression of viral silencing suppressor (VSS) proteins, which can interfere with one or more steps/factors of plant miRNA biogenesis/action. To test this idea, *35S-P19* and *35S-P0* transgenes encoding the VSSs P19 and P0 respectively were transformed into *Arabidopsis* and multiple transformants were obtained for both constructs. Most *35S*-*P19* transformants displayed reduced rosette sizes, indicating that *P19* expression perturbs *Arabidopsis* development (Fig. 5a), a finding previously observed [30]. However, these rosettes displayed no obvious leaf-curling, suggesting that *35S*-*P19* expression was not strongly perturbing miR159 function. By contrast, many *35S-P0* transgenic plants developed severe morphological abnormalities, which were characterized by a reduced rosette size and curled leaves (Fig. 5a). These abnormalities were consistent with what had been previously reported for this *35S-P0* transgene [31], having characteristics similar to that of *mir159ab* rosettes and thus were further investigated.

First, the *35S-P0* primary transformants were grouped into four classes, based on the severity of rosette defects (Fig. 5b). Next, the *P0* transcript level was measured in each class and was found to strongly correlate with the severity of morphological abnormalities (Fig. 5c), suggesting the P0-induced phenotypes are dose-dependent. To determine whether these phenotypes were potentially due to inhibition of miR159 function, *MYB33* and *MYB65* transcript levels were measured by qRT-PCR. With the exception of Class I (wild-type looking phenotype), mild increases (1–3 fold) of *MYB33* and *MYB65* transcript levels were observed in all other *35S-P0* classes, positively correlating with the severity of abnormalities and the level of *P0* transcript (Fig. 5c). However, although increases in *CP1* mRNA levels also positively correlated with both the *P0* and *MYB33*/*65* transcript levels (Fig. 4c), the fold change of *CP1* mRNA level was much lower than that observed in *mir159ab*, both in this study (Fig. 4b) and in previous reports [15]. This suggests that perturbation of miR159 function by P0 expression is mild and de-regulation of *MYB33*/*65* may not be strongly impacting the phenotype of the *35S*-*P0* plants.

To investigate this possibility, the *35S*-*P0* transgene was transformed into a loss-of-function *myb33.myb65* mutant [*35S*-*P0*(*myb33.myb65*)] and grown alongside *35S-P0* Col transformants [*35S*-*P0*(Col)]. The *35S-P0*(*myb33.myb65*) transformants developed similar phenotypes to those of *35S-P0*(Col) and could be grouped into the same phenotypic classes (class I, II, III and IV as shown in Fig. 5b). Moreover, qRT-PCR data demonstrated that the *P0* transcript levels were similar in comparable *35S-P0*(Col) and *35S-P0*(*myb33,myb65*) phenotypic classes (Fig. 5d). This finding indicated that similar P0 expression levels triggered similar phenotypic defects in both Col and *myb33.myb65* plants. Hence, these P0-induced phenotypes are largely *MYB33* and *MYB65* independent, and not related to the mild increase of *MYB33* and *MYB65* mRNA levels in *35S-P0*(Col). This agreed with the weak induction of *CP1* (Fig. 5c). Together, these data suggest that P0 expression is unable to perturb miR159 sufficiently to result in strong de-repression of *MYB33/65* expression.

### The response of a *myb33.myb65* mutant to Turnip Mosaic Virus is indistinguishable from wild-type

The failure of the transgenically expressed VSSs to strongly inhibit miR159 function may relate to their expression levels, which can be very high during viral infection [32]. Thus, to further investigate the possibility of perturbing miR159 function with a biotic stress, *Arabidopsis* was infected with *Turnip Mosaic Virus* (TuMV) that contains the VSS HELPER COMPONENT-PROTEINASE (HC-Pro), which sequesters sRNA duplexes [33, 34]. TuMV inoculations were made by infecting two leaves of three week-old wild-type (Col) plants, followed by two weeks of post-inoculation growth at 21 °C, followed by one week at 15 °C. This lower growth temperature was used as there is evidence that it promotes viral infections [35–37].

**Fig. 6:**
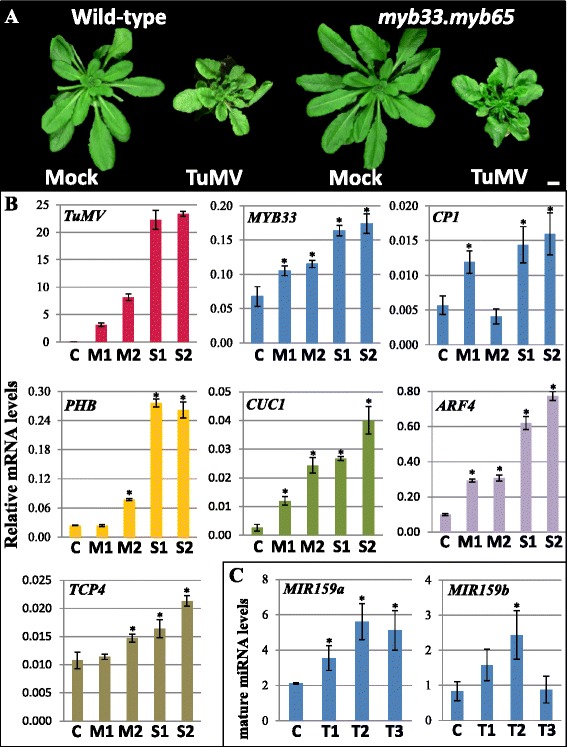
TuMV infection does not appear to strongly perturb miR159 silencing of *MYB33*/*65*. **a** Morphological comparison between TuMV-infected Col and *myb33.myb65* rosettes (21-day-post infection). Plants were inoculated with either Na_2_PO_4_ (mock) or TuMV. **b** qRT-PCR analysis of relative mRNA accumulations in rosettes with TuMV-symptoms being classified as either mild (M) or severe (S). All mRNA levels were normalized to *CYCLOPHILIN*. Error bars represent the SD of three technical replicates. **c** Analysis of mature miR159 levels in three TuMV-infected rosettes, T1-T3. The miR159 levels were normalized to *sno101*. Values are the mean of three technical replicates with error bars representing the SD

Three weeks post-inoculation, the infected rosettes developed symptoms including inhibited growth, upwardly-folded and twisted leaves, and exaggerated serrations of leaf edges and accelerated senescence (Fig. 6a). These symptoms vary in severity, which could be approximated as mild or severe with respect to the rosette size (Additional file 4: Figure S4). To explore the impact of TuMV infection on the miR159-*MYB* pathway, transcript levels of *TuMV*, *MYB33* and *CP1* were analysed in the TuMV-infected wild-type rosettes by qRT-PCR in two plants displaying mild symptoms and two plants displaying severe symptoms. First, analysis found that *TuMV* RNA accumulated to higher levels in the rosettes classified with severe symptoms, suggesting different levels of viral infection (Fig. 6b). Correlating with these *TuMV* transcript levels were *MYB33* mRNA levels that were higher (~2.5 fold) in the TuMV-infected plants compared with uninfected controls (Fig. 6b). Consistent with possible *MYB33* de-regulation, *CP1* mRNA levels had increased (3–4 fold) in most of these infected rosettes. Generally, the abundance of mature miR159a/b were found to accumulate to higher levels in TuMV-infected rosettes (Fig. 6c), consistent with the role of HC-Pro in sequestrating sRNA duplexes, so an increased miR159 abundance likely reflects an accumulation of sequestered miR159 [38]. Although all these data suggest that viral infection can inhibit miR159, given the weak induction of *CP1* in most infected plants, this would predict that *MYB33/65* has only been weakly de-repressed.

To gauge the impact of TuMV infection on other miRNAs families, the mRNA levels of the canonical miRNA targets *PHABULOSA* (*PHB*; miR165/166 target), *CUP-SHAPED COTYLEDON 1* (*CUC1*; miR164 target), *AUXIN RESPONSE FACTOR 4* (*ARF4*; miR390 target) and *TCP4* (miR319 target) were measured by qRT-PCR. The mRNA levels of *PHB*, *CUC1* and *ARF4* were found to increase (8–15 fold) in the rosettes showing severe TuMV symptoms (Fig. 6b). These were generally higher fold-increases than that of *MYB33* (~3 fold, Fig. 6b). Only *TCP4* (~2 fold) had mRNA levels increases similar to *MYB33*, possibly due to the low expression of miR319 in the rosette [39, 40]. Therefore, these data suggest that in comparison with miR159, other miRNA pathways might be more susceptible to de-regulation by TuMV infection, making a stronger contribution to the observed symptoms.

Finally, to determine the contribution of *MYB33*/*65* de-regulation to the manifestation of TuMV symptoms, a comparison of TuMV-infected Col and *myb33.myb65* plants was performed. Both TuMV-infected Col and *myb33.myb65* plants developed similar abnormal leaves and rosettes that appeared indistinguishable from one another (Fig. 6a). Together, all this data suggest that, although TuMV can inhibit miR159, it may do only weakly, being of no major physiological consequence for the plant in response to viral infection.

## Discussion

### The miR159-*MYB33/65* pathway has no major impact on rosette development or abiotic stress response

In Arabidopsis, several conserved miRNA families (e.g. miR156, miR164 and miR165/166) control rosette development via regulation of their targets in specific spatiotemporal manners, impacting major leaf developmental traits such as phase change, leaf polarity and serration [41–44]. In contrast, miR159 appears constitutively expressed throughout rosette development, both spatially and temporally, where it constantly represses *MYB33*/*65* expression as *CP1* mRNA levels remained low. This extends our previous data showing that *MYB33* and *MYB65* are strongly repressed in Arabidopsis vegetative tissues [15, 16]. From our data we cannot rule out that MYB33/65 are expressed transiently or in a subtle cell type(s), where they may subtly impact development. However, it would appear that these genes are not playing a dominant role in Arabidopsis rosette developmental ontogeny, at least under standard laboratory growth conditions. Such a case is similar in rice, where the absence of *GAMYB* and *GAMYB-like* transcripts in vegetative tissues and the lack of obvious vegetative developmental abnormalities of a *gamyb* mutant, implies these genes play no major role in vegetative development [2, 7].

Despite the lack of clear function, the pathway remains active throughout rosette development as inducible inhibition of miR159 could induce leaf curling. Therefore, this led to the hypothesis that the miR159-*MYB33*/*65* pathway may be responsive to an abiotic stress, where if miR159 is repressed, de-repression of *MYB33/65* may possibly result in physiological/developmental outcomes that contribute to stress tolerance. Supporting such a possibility are numerous studies reporting the alteration of miR159 levels in response to stress, implicating miR159 as a general stress-responsive miRNA [4, 13, 19–23]. Despite this, we could find no evidence to indicate that miR159 becomes repressed under similar stress conditions, or changes in response to stress-related hormones such as ABA. Again, we cannot rule out that in certain cell types or under other stress conditions, or a combination of stress conditions, the miR159-*MYB33*/*65* pathway does play a role. For example, the *myb33.myb65* mutant has been shown to respond differently to wild-type after 4 h at 44 °C [13]. However, from our data, it appears that no major functional impact in the response to the tested stresses can be ascribed to the miR159-*MYB* pathway, as there was no overt difference between wild-type and the *mir159ab.myb33.myb65* mutant in response to these stresses. This also suggests that functionally relevant miR159 regulation of other targets is improbable as the absence of miR159 in the *mir159ab.myb33.myb65* mutant does not make a major difference under the tested stresses. Therefore, we propose that many of the fluctuations in miR159 levels observed during stress may have no major impact of functional consequence.

### Expression of VSSs fails to strongly inhibit miR159 repression of *MYB33*/*65*

Consequently, we shifted our attention to biotic stresses, including viruses that express silencing suppressors (VSS) that could repress miR159. As many viruses can result in symptoms resembling *mir159ab*-like phenotypes, such as Tomato Leaf curl virus that causes leaves to curl upwards to which miR159 has been linked [25], we explored whether the transgenic expression of VSSs or infection with viruses containing VSSs could perturb miR159. However, in these experiments, all our data indicates that miR159 silencing of *MYB33*/*65* is not perturbed enough for this pathway to play a major role in response to such biotic stresses. For instance, similar P0 expression levels in *35S-P0*(Col) and *35S-P0*(*myb33myb65*) plants triggered symptoms of indistinguishable severity (Fig. 5d), indicating that the up-regulated expression of MYB33/65 in *35S-P0*(Col) was not a major factor in the observed P0-induced symptoms. Additionally, TuMV induced defects in Col and *myb33.myb65* plants appeared phenotypically indistinguishable (Fig. 6a), consistent with the marginal increased levels of *MYB33*/*65* and *CP1* in TuMV infected Col plants (Fig. 6b), again suggesting that perturbation of miR159-mediated regulation of *MYB33*/*MYB65* plays no major role in TuMV symptoms. Both experiments suggest miR159 silencing of *MYB33*/*65* is robust; given the morphological defects of the *35S-P0* plants or the transcript profiling in the TuMV challenged plants, it was likely that other endogenous miRNA pathways were strongly inhibited contributing in the observed morphological defects.

However, Du et al. [24] reported a possible causative role of miR159 in disease symptoms induced by a *Cucumber Mosaic Virus* (i.e. Fny-CMV), as they compared the Fny-CMV infected Col and *myb33.myb65*, showing phenotypic evidence that the infected Col plants displayed more deformation of the upper, young systemically infected leaves. Based on this, they concluded that miR159 contributes to Fny-CMV induced symptoms [24]. Therefore, the possibility cannot be excluded that VSSs differentially perturb the different miRNA families, and that a VSS exists that preferentially perturbs miR159, like the identified viral impact on miR168 accumulation [37].

## Conclusions

Hence, despite our efforts, and a large body of previous work examining miR159 expression in Arabidopsis rosettes, we have been unable to define a major role for the miR159-*MYB33*/*65* pathway in the rosette. What is clear is that miR159 robustly represses *MYB33*/*65*, where neither P0 and P19 VSSs nor a range of stresses appear able to reduced miR159 sufficiently to enable de-regulation of *MYB33*/*MYB65* expression to result in an obvious phenotype impact in response to the stress. With regards to general inhibitors of the miRNA pathway, such as VSSs, it seems other miRNA systems are more sensitive to these inhibitors than miR159. It would appear that an inhibitor that is specific to miR159 would be needed to result in activation of the *MYB* pathway. Curiously, in Arabidopsis seeds, miR159 silencing of *MYB33*/*65* appears weak relative to rosette tissue [16], suggesting the presence of such an inhibitor, or another factor that controls silencing efficacy, may exist.

Although the highly conserved miR159-*MYB* pathway may have a regulatory role in the vegetative tissues of other plant species, here our data re-enforces the notion, that in Arabidopsis, the predominant function of miR159 is to restrict the expression of *MYB33* and *MYB65* to seeds and anthers. Interestingly, other *GAMYB-like* genes in Arabidopsis, such as *MYB101*, are predominantly transcribed in seeds and anthers, and this is also appears the case for *GAMYB* in cereals [2, 13], both of which strongly contrast the apparent ubiquitous transcription of *MYB33*/*65* in *Arabidopsis*. Given that there are multiple *GAMYB-like* genes required for different steps of male development in Arabidopsis [9, 45, 46], during the duplication and divergence of *MYB33*/*65*, these genes appear to have acquired this near constitutive transcriptional domain. As the activity of *MYB33* and *MYB65* promotes male fertility, there would be strong selection pressure for their strong expression. Hence, we speculate this may result in strong transcription not only in the anther, but also in vegetative tissues. Any negative impact of unnecessary *MYB33*/*65* transcription in vegetative tissues (followed by the required miR159 silencing), would be vastly outweighed by enhanced male fertility. Indeed, although it could be considered that this miR159-*MYB33*/*65* “futile” pathway may be energetically wasteful, there appears no obvious difference between wild-type and *mir159ab.myb33.myb65* rosettes, and so such an energy investment may be not be large enough to confer a selective disadvantage. Therefore, we speculate, that if a gene is miRNA-regulated, there may be less pressure on cis-acting promoter elements to define its required spatial/temporal transcription pattern, as post-transcriptional regulation by miRNAs provides an alternative mechanism to achieve the required protein expression.

## Methods

### Plant materials and growth conditions

*Arabidopsis thaliana* ecotype Columbia-0 (Col-0) was used in all experiments and is referred to as wild type. The following mutants were described previously and represent T-DNA insertional loss-of-function mutants: *mir159a*, *mir159ab* [10] and *myb33.myb65* [9]. The transgenic lines *MIR159b:GUS* and *mMYB33:GUS* were previously generated and described [10]. Seeds were either sown on soil (Debco Plugger soil mixed with Osmocote Extra Mini fertilizer at 3.5 g/L) or on agar plates containing 0.5X *MS* (Murashige and Skoog, 2.2 g/L), and stratified at 4 °C overnight in the dark. Plants were grown in 21 °C growth cabinets under either long day (LD) (16 h light/8 h dark, fluorescent illumination of 150 μmol m^−2^ s^−1^) or short day (SD) photoperiod (8 h light/16 h dark, fluorescent illumination of 150 μmol/m^2^/sec). For stress treatments, plants were grown side by side in soil for two weeks in a 21 °C growth chamber (a LD photoperiod was applied throughout the treatment if not otherwise specified), and then transferred into a 4 °C growth room (low-temperature treatment), or high temperature (32 °C day/28 °C night), or high light intensity (~500 μmol m^−2^ s^−1^), or provided with ~800 mL tap water per litre soil per two weeks (drought stress). One tray (30 plants) were used for each treatment. For TuMV infection, TuMV-infected tobacco leaves were ground in 5 mM sodium phosphate buffer (pH 7) containing silicon carbide, which were used to mechanically inoculate two largest leaves of three-week-old Arabidopsis rosettes.

### Generation of transgenic *Arabidopsis*

Gateway compatible entry vectors harbouring P0 and P19 were sub-cloned into the Gateway compatible destination vector pMDC32 containing the 35S promoter [47] via a Gateway LR Clonase reaction according to the manufacturer’s instructions (Invitrogen). For inducible expression of *MIM159*, the destination vector pMDC7 containing the 17-β-estradiol inducible transcription activator XVE was used [28]. The LR reaction mixture was transformed into *E. coli* Alpha-Select Gold Efficiency competent cells (Bioline) by heat shock with bacteria grown on LB plates containing the corresponding selection antibiotics (50 μg/mL Kanamycin for pMDC32 based vectors, 50 μg/mL spectinomycin for pMDC7 based vectors).

All expression vectors were transformed into *Agrobacterium tumefaciens* strain GV3101 by electroporation [48], and incubated on LB plates containing Rifamycin (50 μg/mL), Gentamicin (25 μg/mL), and either Kanamycin (50 μg/mL for pMDC32 based vectors) or spectinomycin (50 μg/mL for pMDC7 based vectors). *Agrobacterium* was prepared in infiltration medium containing 5 % sucrose and 0.03 % Silwet L-77 and used to transform Arabidopsis via the floral dip method [49]. For estradiol induction of the *XVE-MIM159* transgene, three-week primary transformants were sprayed with either 10 μM 17-β-estradiol (inducer) or 10 μM dimethyl sulfoxide (DMSO, solution to dissolve 17-β-estradiol). The treatment was repeated once every three days for two weeks.

### PCR genotyping of T-DNA insertional alleles

DNA extractions were performed using the Edward preparation method (Edwards et al., 1991). Then, PCR was carried out using Platinum® Taq DNA Polymerase (Invitrogen) in a 20 μL reaction volume. 2 μL of genomic DNA was used for each PCR, with final primer concentration at 0.2 μM. PCR conditions used were one cycle of 94 °C/ 2 min; 30 cycles of 95 °C/30 sec, 60 °C/30 sec, 72 °C/1–2 min; one cycle of 72 °C for 5 min. 10 μL of each PCR reaction was analysed on a 1 % agarose gel by electrophoresis. Primer sequences used for genotyping are those that have been previously reported [9, 10].

### Quantitative Real-time PCR (qRT-PCR) and Taqman microRNA analysis

TRIzol® (Invitrogen) was used for RNA extraction as has previously been reported (Allen at al., 2010). For qRT-PCR, 30–50 μg of total RNA was treated with 25 μL of RQ1 RNase-Free DNase in a 100 μL reactions following the manufacturer’s protocol (Promega), with the addition of 2.5 μL of RNaseOut™ Recobinant RNase Inhibitor (Invitrogen). The digested samples were purified with the Spectrum^IM^ plant Total RNA Kit (Sigma Aldrich) to remove digested DNA fragments. cDNA synthesis was carried out using SuperScript® III Reverse Transcriptase (Invitrogen) and oligo dT primers according to the manufacturer’s instructions. For qRT-PCR, Platinum® Taq DNA Polymerase (Invitrogen) with SYB Green (Sigma) and dNTPs (Fisher Biotec) added was used as a master mix. 10 μL of each cDNA sample was added to 9.6 μL of SYB/Taq master mix with 0.4 μL of forward and reverse primers at 10 μmol each. All qRT-PCR reactions were carried out on a Rotor-Gene Q real-time PCR machine (Qiagen) using the following cycling: one cycle of 95 °C/5 min; 45 cycles of 95 °C/15 sec, 60 °C/15 sec, 72 °C/20 sec. *CYCLOPHILIN* (At2g29960) or *UBIQUITIN* (At4g05320) was used to normalize mRNA levels using the comparative quantitation program in the Rotor-Gene Q software provided by Qiagen, and these relative measurements are presented.

For determining mature miR159 levels, TRIzol purified RNA sample was directly subjected to TaqMan MicroRNA Assays (Applied Biosystems) following the protocol described by Allen et al. [17]. This assay used 10 ng of each RNA sample to perform the retro-transcription, with the use of a TaqMan MicroRNA Reverse Transcription kit (Applied Biosystems), and each reaction included the stem-loop RT primers for both the miR159a (or miR159b) and the normalization sRNA *sno101*. Then, each cDNA was assayed in triplicate using the Rotor-Gene Q machine using the cycling conditions described above. The Expression of miR159a (or miR159b) were normalized with *sno101* using the comparative quantitation analysis program in the Rotor-Gene Q software and these relative measurements are presented. Values presented for both mRNA and miRNA measurements are the mean of three technical replicates (three cDNA syntheses) of one biological replicate (one RNA isolation of multiple plants unless otherwise stated). The value for each technical replicate (cDNA reaction) is determined by assaying the individual technical replicate three times. Experiments were then independently repeated at least once (independent biological replicates) to confirm findings. Unpaired T-tests were applied to values to determine if differences were statistically different.

### GUS staining

GUS staining was performed on rosette tissues using the method described by Jefferson [50] with the following modifications: 1) Rosette tissues were collected and fixed with 90 % acetone for 20 min at room temperature, followed by a 30 min vacuum infiltration with GUS staining buffer 1 (50 mM Na phosphate buffer, pH 7.2, 0.2 % Triton X-100, 2 mM potassium ferricyanide and 2 mM potassium ferrocyanide). 2) Histochemical reactions were performed by a 30 ~ 60 min vacuum infiltration with staining buffer 2 (staining buffer 1 plus 2 mM X-gluc) and then an overnight incubation at 37 °C. The staining buffer was removed by successive washes with 20 %, 50 %, 70 % and 90 % ethanol (1 h per wash), and the cleared tissues were photographed using an Olympus Dissecting Microscope OLYMPUS SZX2-ILLK (Tokyo, Japan) (for 10 ~ 20-day-old seedlings).

## Additional files

Additional file 1: Figure S1.
*CP1* transcript level tightly correlates with MYB33/65 expression. (A) Representative rosette phenotypes of five-week-old *mir159ab* plants carrying different combinations of heterozygous or homozygous T-DNA insertion *myb33/myb65* alleles. The F3 progenies were numbered in order as the genotyping was performed, and one numbered plant of every confirmed genotype was selected to represent the rosette phenotype. (B) qRT-PCR analysis of *CP1* mRNAs in five-week-old rosettes of different genotypes. The numbers along the x-axis correspond to the numbered plants shown in A. Transcript levels were normalized to *CYCLOPHILIN*, where values are the mean of three technical replicates with error bars representing the SD. (PPTX 1367 kb) Additional file 2: Figure S2. Time-course of miR159a level throughout rosette development. The relative miRNA levels were measured in rosettes approximately every 10 days throughout its development. The miR159 levels were normalized to *sno101*. Values are the mean of three technical replicates with error bars representing the SD. Significant differences in values from the previous measurement are indicated with an _*_, as determined by the Students *T*-test. (PPTX 48 kb) Additional file 3: Figure S3. The normalizing RNA, *sno101*, appears to increase under high temperature. Cycling output for *sno101* and miR159a measurements showing in the high-temperature measurements the increase in *sno101* output. MiR159a levels appear most affected by cold. (PPTX 398 kb) Additional file 4: Figure S4. Morphological impact of TuMV infection. Representative classification of symptom severities among TuMV-infected rosettes. (PPTX 1545 kb)

## Abbreviations

*ARF4*: *AUXIN RESPONSE FACTOR 4*
GUS: β-glucuronidase
CMV: *Cucumber Mosaic Virus*
*CUC1*: *CUP-SHAPED COTYLEDON 1*
*CP1*: *CYSTEINE PROTEINASE1*
DMSO: Dimethyl sulfoxide
GA: Gibberellin
HC-Pro: HELPER COMPONENT-PROTEINASE
*PHB*: *PHABULOSA*
PCD: Programmed cell death
TuMV: *Turnip Mosaic Virus*
VSS: Viral silencing suppressor
