# Supplementary material for: Ubiquitous miR159 repression of MYB33/65 in Arabidopsis rosettes is robust and is not perturbed by a wide range of stresses
Source: BMC Plant Biol. 2016 Aug 19;16:179. doi: 10.1186/s12870-016-0867-4 (PMC4992245; doi:10.1186/s12870-016-0867-4)
Supplement: Additional file 1: Figure S1. — CP1 transcript level tightly correlates with MYB33/65 expression. (A) Representative rosette phenotypes of five-week-old mir159ab plants carrying different combinations of heterozygous or homozygous T-DNA insertion myb33/myb65 alleles. The F3 progenies were numbered in order as the genotyping was performed, and one numbered plant of every confirmed genotype was selected to represent the rosette phenotype. (B) qRT-PCR analysis of CP1 mRNAs in five-week-old rosettes of different genotypes. The numbers along the x-axis correspond to the numbered plants shown in A. Transcript levels were normalized to CYCLOPHILIN, where values are the mean of three technical replicates with error bars representing the SD. (PPTX 1367 kb) [file 12870_2016_867_MOESM1_ESM.pptx]

## Slide 1
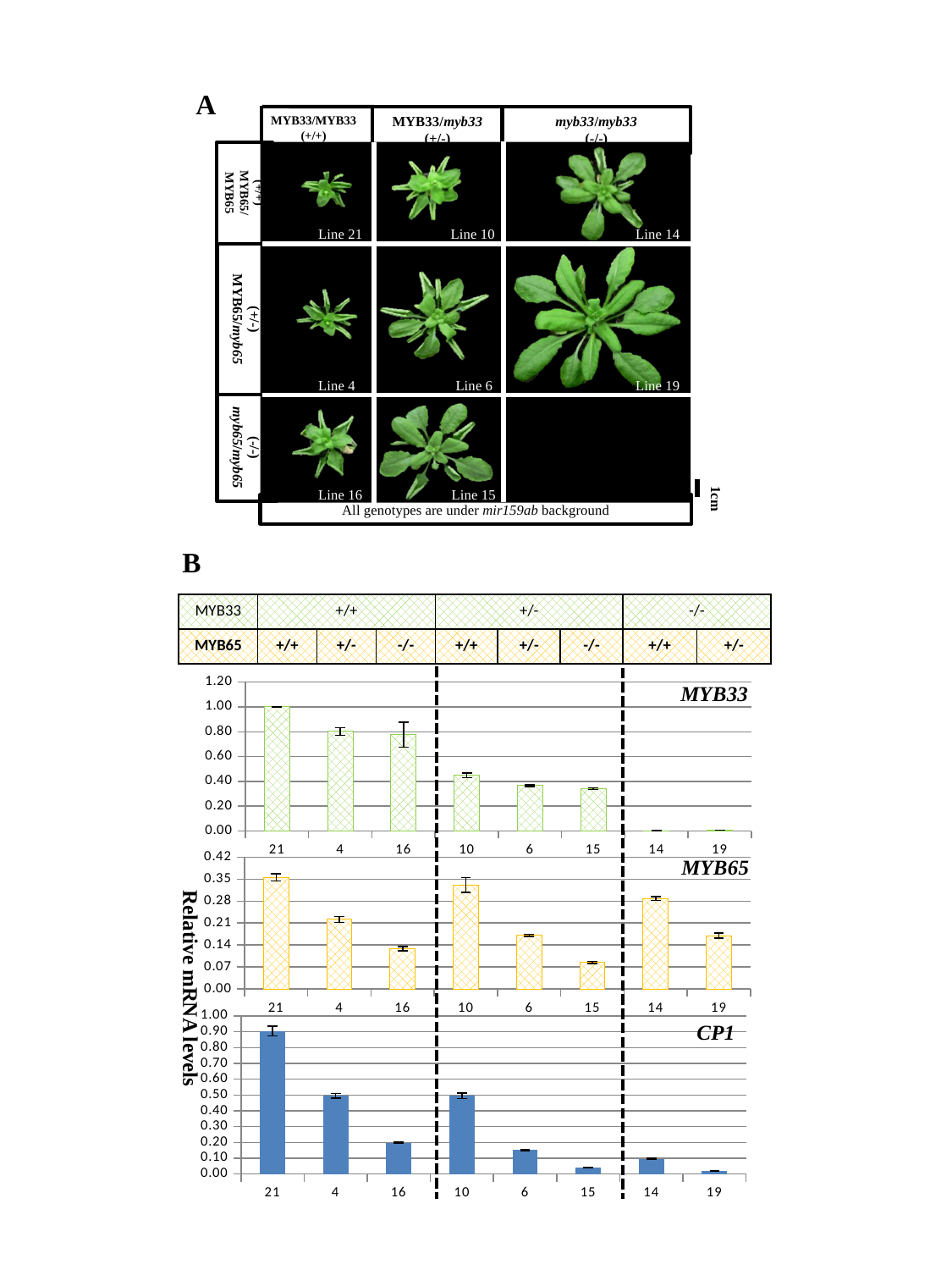

A
MYB33/MYB33
(+/+)
myb33/myb33
(-/-)
MYB33/myb33
(+/-)
1cm
(+/+)
MYB65/MYB65
(+/-)
MYB65/myb65
(-/-)
myb65/myb65
All genotypes are under mir159ab background
Line 10
Line 21
Line 14
Line 4
Line 19
Line 6
Line 15
Line 16
B
| MYB33 | +/+ | | | +/- | | | -/- | |
| --- | --- | --- | --- | --- | --- | --- | --- | --- |
| MYB65 | +/+ | +/- | -/- | +/+ | +/- | -/- | +/+ | +/- |
### Chart: MYB33
| Category | |
|---|---|
| 21 | 1.0 |
| 4 | 0.8029999999999999 |
| 16 | 0.7746666666666666 |
| 10 | 0.449 |
| 6 | 0.365 |
| 15 | 0.3413333333333333 |
| 14 | 0.00197 |
| 19 | 0.005513333333333333 |
### Chart: MYB65
| Category | MYB65 |
|---|---|
| 21 | 0.35566666666666674 |
| 4 | 0.22166666666666668 |
| 16 | 0.128 |
| 10 | 0.3316666666666666 |
| 6 | 0.17066666666666666 |
| 15 | 0.08446666666666665 |
| 14 | 0.2886666666666666 |
| 19 | 0.17 |Relative mRNA levels
### Chart:
| Category | CP1 |
|---|---|
| 21 | 0.9053333333333334 |
| 4 | 0.49433333333333335 |
| 16 | 0.19833333333333333 |
| 10 | 0.49499999999999994 |
| 6 | 0.15133333333333332 |
| 15 | 0.0391 |
| 14 | 0.09536666666666667 |
| 19 | 0.01786666666666667 |
