# Supplementary figures and images for: Ubiquitous miR159 repression of MYB33/65 in Arabidopsis rosettes is robust and is not perturbed by a wide range of stresses
Source: BMC Plant Biol. 2016 Aug 19;16:179. doi: 10.1186/s12870-016-0867-4 (PMC4992245; doi:10.1186/s12870-016-0867-4)

## Slide 1
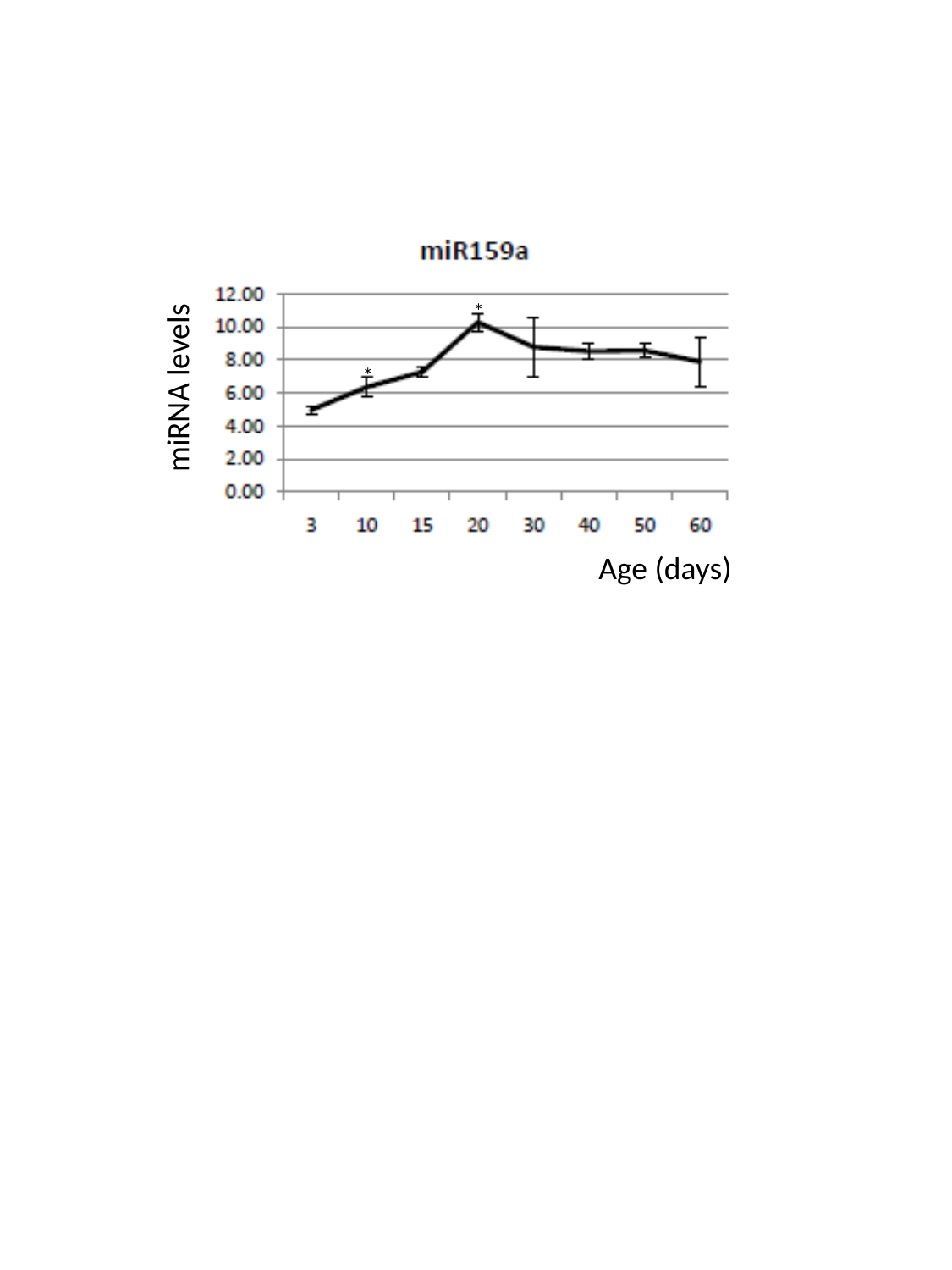

*
*
miRNA levels
Age (days)

Supplement: Additional file 2: Figure S2. — Time-course of miR159a level throughout rosette development. The relative miRNA levels were measured in rosettes approximately every 10 days throughout its development. The miR159 levels were normalized to sno101. Values are the mean of three technical replicates with error bars representing the SD. Significant differences in values from the previous measurement are indicated with an *, as determined by the Students T-test. (PPTX 48 kb) [file 12870_2016_867_MOESM2_ESM.pptx]

## Slide 1
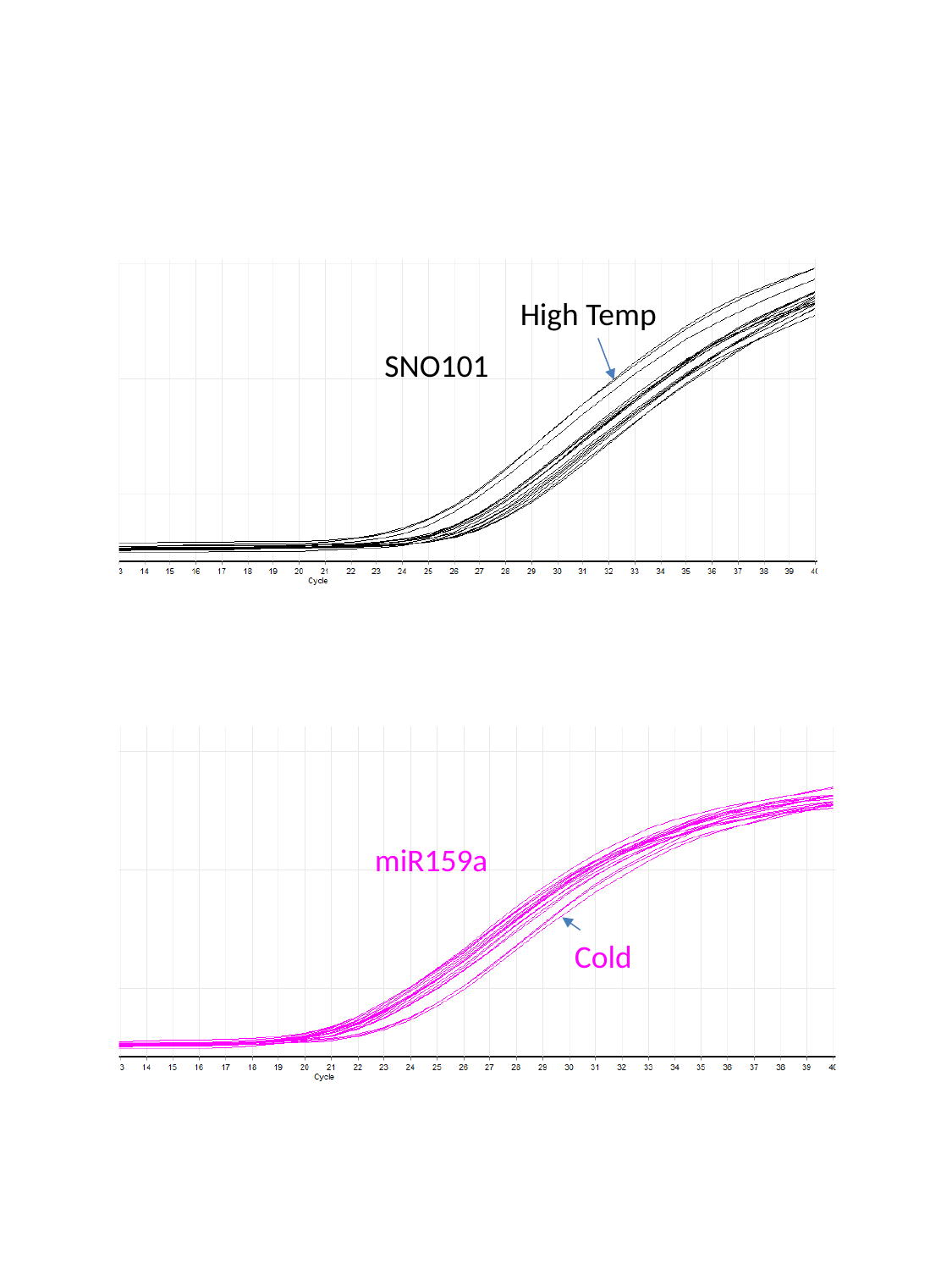

High Temp
SNO101
miR159a
Cold

Supplement: Additional file 3: Figure S3. — The normalizing RNA, sno101, appears to increase under high temperature. Cycling output for sno101 and miR159a measurements showing in the high-temperature measurements the increase in sno101 output. MiR159a levels appear most affected by cold. (PPTX 398 kb) [file 12870_2016_867_MOESM3_ESM.pptx]

## Slide 1
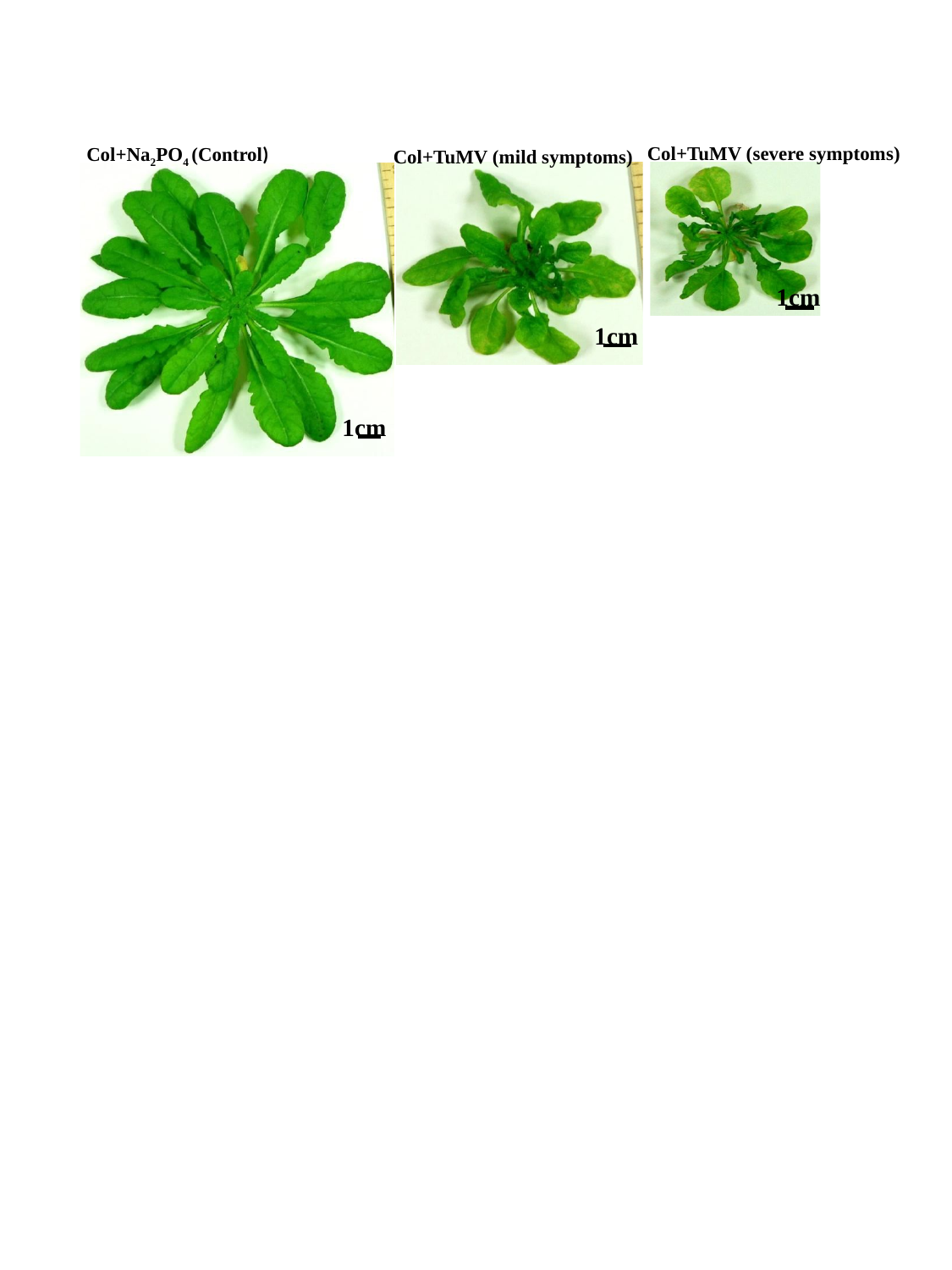

Col+TuMV (severe symptoms)
Col+Na2PO4 (Control)
Col+TuMV (mild symptoms)
1cm
1cm
1cm

Supplement: Additional file 4: Figure S4. — Morphological impact of TuMV infection. Representative classification of symptom severities among TuMV-infected rosettes. (PPTX 1545 kb) [file 12870_2016_867_MOESM4_ESM.pptx]
